# Yellow Fever in the South West

**Published:** 1840-01

**Authors:** 


### YELLOW FEVER IN THE SOUTH-WEST

MOST of our readers, are aware, that the past autumn has been calamitously signalized by the diffusion and mortality of this endemic of the Delta of the Mississippi, and the adjoining shores of the Gulph of Mexico. Those regions have, perhaps, at no previous time since their settlement by civilized man, been so severely and extensively scourged. From Florida to Texas along the Gulf, and up the river to Arkansas and Mississippi, most of the principal towns and many villages have been invaded. Within that extensive region, there are many able physicians who have been in the midst of the pestilence, on which, as to its ætiology and treatment they must have made a variety of new and important observations. We solicit for our journal the experience ef the whole, and hope in successive numbers, to be able to present a full history of the epidemic. Meanwhile (although not rigorously professional) we offer to our readers, the following extract of a letter from the intelligent wife of a respectable physician in Mobile, giving an account of the ravages of the pestilence in that desolated city:

"Your letter of July 26th, reached me in August, just at the period when the banner of death was unfurled over our devoted city. A letter from you, my dear friend, is ever welcome, but your last letter was doubly so.

"For it came when my bosom was lonely and dark,
"Like the beacon's glad blaze to the mariner's bark,
"Like the sunlight that breaks through the clouds of the storm."

Sorrow and affliction were every where round me. Dr. L. was very ill of the Yellow Fever; and each day, tidings came to us of the death of friends. I am happy to tell you, my friend, that none of our immediate family have fallen victims to the fell destroyer. Dr. L. recovered, after a severe illness, and soon resumed the duties of his profession. Neither my father, mother, brother nor myself, has suffered from the fever. We are perchance the only persons out of 10,000 who have escaped a touch of the malady. I doubt not our exemption arises from our long residence on the Gulf of Mexico, where the yellow fever especially delights to dwell.

In June we had removed from Mobile to our country place, three miles from the city. But the country has been as much afflicted as the city; and pure air and verdant trees failed to insure health to those who fled from the pestilence. I am persuaded the annals of history offer no parallel to the affliction of Mobile, since the midsummer sun beamed upon us. The description of the plague of Athens, in the time of Pericles, or of the plague in London, in the seventeenth century, is a faithful picture of the yellow plague of this place, which has sent its hundreds to the silent tomb.

Words have not the power to shadow forth the fearful scenes of this season. In wretched holes have been found the lifeless bodies of husband and wife, with an infant striving to draw nourishment from the dead mother's cold bosom. Whole families were swept off as by an avalanche. The strong man, in his highest health, was struck down in a few days. The delicate female, the young child, the aged, the rich and the poor, those whose dwelling was the splendid mansion, and those whose lodgings were the cold ground, all, all, alike has the "insatiate archer death" numbered as his victims. Oh! my soul sickens at the anguish crowded into a few months.

When autumn came, hope whispered that its frosts would stay the steps of the destroyer. Alas! the very elements are against us. The earth is parched; no rains fall; and the sun has all the heat of tropical climes. Each day, I may say each hour, we hear of some valuable life that has passed away — some father whose large family looked in helplessness to him for their daily bread; some fond mother, whose little ones were supported by her daily toil. May God in his mercy, look down upon the new-made orphans of these fatal months!" D.

